# Normobaric oxygen paradox and the microcirculation in the critically ill patient: a prospective observational study

**DOI:** 10.1186/cc13358

**Published:** 2014-03-17

**Authors:** A Donati, E Damiani, AT Colesnicenco, E Montesi, S Ciucani, A Carsetti, P Pelaia

**Affiliations:** 1 Università Politecnica delle Marche, Ancona, Italy

## Introduction

The normobaric oxygen paradox (NOP) is a recent concept that postulates the use of intermittent hyperoxia to stimulate erythropoietin (EPO) production [1]. Hyperoxia increases oxygen free radicals and may lead to endothelial damage and vasoconstriction [2]. We evaluated the microvascular response to transient hyperoxia and its effects on EPO production.

## Methods

Six patients with hemodynamic stability and mechanically ventilated with FiO_2 _<50% were included in this prospective observational study. Patients underwent a 2-hour period of hyperoxia (FiO_2 _100%). The sublingual microcirculation (sidestream dark-field imaging (SDF)) was evaluated at baseline (t0), 2 hours after hyperoxia (t1), and 2 hours after return to basal FiO_2 _(t2). SDF monitoring was continuously performed also during the variation of FiO_2 _for 2 minutes. EPO levels were assayed at baseline and for 2 days.

## Results

**Figure 1:**
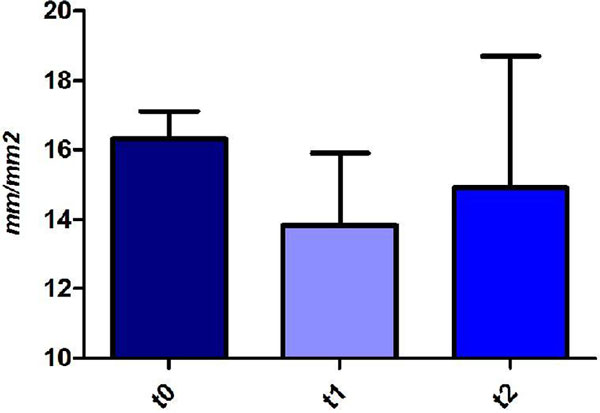
**TVD and NOP**.

An early vasoconstriction and a trend towards total vessel density (TVD) reduction were observed at t1 (Figure 1). The TVD tended to increase without returning to baseline levels at t2. EPO increased in four patients out of 6 (*P *= NS). A negative correlation was found between the change in TVD after hyperoxia (t1 - t0) and the change in EPO (*r *= -0.88, *P *= 0.03).

## Conclusion

Hyperoxia leads to vasoconstriction that seems to be reversible at hyperoxia cessation. Further data are needed to verify the efficacy of the NOP in stimulating erythropoiesis in the critically ill. There might be a relation between hyperoxia-induced reduction in vessel density and the EPO increase.

## References

[B1] Balestra C Germonpré P Poortmans JR Marroni A J Appl Physiol 2006 100 512 518 10.1152/japplphysiol.00964.2005 16239610

[B2] Tsai AG Cabrales P Winslow RM Intaglietta M Am J Physiol Heart Circ Physiol 2003 285 H1537 H1545 12805029 10.1152/ajpheart.00176.2003

